# Hépatite virale B et insuffisance rénale: prévalence et facteurs associés au Centre National Hospitalier et Universitaire de Cotonou

**DOI:** 10.11604/pamj.2018.31.121.16498

**Published:** 2018-10-17

**Authors:** Jean Séhonou, Aboudou Raïmi Kpossou, Taofick Oyétoundé Amanda, Comlan N’dehougbea Martin Sokpon, Rodolph Koffi Vignon, Jacques Vigan

**Affiliations:** 1 Clinique Universitaire d’Hépato-Gastroentérologie, Centre National Hospitalier et Universitaire Hubert Koutoukou Maga (CNHU-HKM), Cotonou, Bénin; 2 Faculté des Sciences de la Santé (FSS), Université d’Abomey-Calavi, Bénin; 3 Clinique Universitaire de Néphrologie-Hémodialyse, Centre National Hospitalier et Universitaire Hubert Koutoukou Maga (CNHU-HKM), Cotonou, Bénin

**Keywords:** Insuffisance rénale, hépatite B, facteurs associés, Renal failure, hepatitis B, associated factors

## Abstract

**Introduction:**

les relations entre les reins et l'hépatite B sont complexes. L'objectif de cette étude était de déterminer la prévalence et les facteurs associés à l'atteinte rénale chez des personnes vivant avec le virus de l'hépatite B (PVVHB) à Cotonou.

**Méthodes:**

il s'agissait d'une étude transversale à visée descriptive et analytique, menée dans le service d'hépato-gastroentérologie du CNHU-HKM de mai à août 2017. Elle avait inclus les patients porteurs d'Ag HBs reçus dans la période. Une insuffisance rénale était retenue si le débit de filtration glomérulaire estimé par la formule du MDRD était en dessous de 90 mL/min/1,73m^2^.

**Résultats:**

105 patients porteurs d'Ag HBs étaient inclus. Parmi eux, 65 (61,9%) étaient sous traitement anti-VHB (dont 62 sous ténofovir). Des 105 patients, 41 avaient une insuffisance rénale (39%). Cette insuffisance rénale était associée à 2 cas d'atteinte tubulaire et 4 cas d'atteinte glomérulaire. La fonction rénale s'était dégradée dans le temps chez 22 patients (21%) et s'était améliorée chez 6 patients (5,7%). En analyse univariée, les facteurs associés à la survenue d'une insuffisance rénale étaient l'âge supérieur à 50 ans (p = 0,0125), la présence d'une hypertension artérielle (p = 0,0037), une fonction rénale initialement altérée (p < 0,0003) et les co-médications (p = 0,0007). Le traitement anti-VHB n'était pas associé à la survenue d'une insuffisance rénale (p = 0,2887).

**Conclusion:**

la prévalence de l'insuffisance rénale chez les PVVHB était élevée (39%). L'âge, l'hypertension artérielle, une insuffisance rénale préexistante et les co-médications étaient identifiés comme des facteurs associés au déclin de la fonction rénale chez les PVVHB.

## Abstract (en)

**Introduction:**

the association between the kidneys and hepatitis B is complex. This study aims to determine the prevalence and factors associated with renal disease in people living with hepatitis B virus (PLHBV) in Cotonou.

**Methods:**

we conducted a cross-sectional, descriptive and analytical study in the Hepatogastroenterology Department at the National University Hospital Center in Cotonou over the period May -August 2017. All the patients with positive hepatitis B surface antigen (HBS Ag) test hospitalized in the study period were included. The diagnosis of renal failure was retained in patients with glomerular filtration rate less than 90 mL/min/1.73 m^2^ (estimated with MDRD Equation).

**Results:**

the study involved 105 patients with positive hepatitis B surface antigen (HBS Ag) test Among them, 65 (61.9%) were under anti-HBV treatment ( 62 of them were under tenofovir ); 41 patients had renal failure (39%) with tubular involvement (2 cases) and glomerular involvement (4 cases). Renal function had got progressively worse over time in 22 patients (21%) and had improved in 6 patients (5.7%). Univariate analysis showed that factors associated with the occurrence of renal failure were: age greater than 50 years (p = 0.0125), high blood pressure (p = 0.0037), initially abnormal renal function (p < 0.0003) and co-medications (p = 0.0007). Anti-HBV treatment wasn't associated with the occurrence of renal failure (p = 0.2887).

**Conclusion:**

the prevalence of renal failure in PLHBV was high (39%). Age, arterial hypertension, pre-existing renal failure and co-medications were identified as factors associated with decline in renal function in PLHBV.

## Introduction

Le déclin de la fonction rénale observé au cours de l'hépatite chronique B peut engager le pronostic vital, s'il n'est pas diagnostiqué et pris en charge. Il est toutefois difficile d'identifier les patients vulnérables qui peuvent avoir une dysfonction rénale car les relations entre le virus de l'hépatite B (VHB) et les reins sont complexes [1]. En effet, d'une part dans l'histoire de la maladie, la présence de mécanismes immunologiques impliquant des antigènes viraux et des anticorps spécifiques anti-VHB serait responsable de plusieurs manifestations extra-hépatiques. Au nombre de ces manifestations, les glomérulonéphrites à VHB (GN-VHB) et les périartérites noueuses (PAN) sont les plus caractéristiques et susceptibles d'induire des lésions rénales [2]. D'autre part un traitement prolongé avec le ténofovir disoproxil fumarate (TDF), a été associé à de rares cas de dysfonction tubulaire rénale proximale survenant généralement après des mois ou des années de traitement. Cette lésion rénale se manifeste le plus souvent par un syndrome de Fanconi avec une insuffisance rénale aiguë et une atteinte tubulaire distale sous la forme d'un diabète insipide néphrogénique [3]. La plupart des études sur le TDF ont été effectuées chez des patients présentant initialement une bonne fonction rénale et le plus souvent exempts de toute comorbidité. Les objectifs de cette étude étaient de déterminer la prévalence de l'atteinte rénale chez les PVVHB suivis au CNHU-HKM de Cotonou et d'identifier les différents facteurs qui y étaient associés.

## Méthodes

Un total de 208 patients avait consulté à la clinique universitaire d'hépato- gastroentérologie du CNHU-HKM de Cotonou depuis que le traitement par TDF y a débuté (de janvier 2012 à août 2017). Notre étude a porté sur les patients de cette cohorte admis de façon consécutive en consultation, qu'ils fussent traités ou non par du TDF ou par de l'Interféron pégylé alpha (IFN-PEG). Etaient inclus dans l'étude les patients ayant donné leur consentement éclairé et dont le dossier médical comportait un bilan minimum pré-thérapeutique avec au moins une créatininémie et une phosphorémie. Etaient exclus de l'étude les patients de la cohorte susmentionnée qui étaient perdus de vue. Les données étaient collectées à l'aide d'une fiche d'enquête comportant des variables sociodémographiques, cliniques, paracliniques et thérapeutiques. Pour ce faire, nous avions procédé à une revue documentaire des dossiers en vue de la sélection des patients répondant à nos critères d'inclusion, avant l'entretien en tête à tête avec les patients. Cet entretien a permis entre autres: d'expliquer les objectifs de l'étude et d'obtenir un consentement éclairé, de compléter les informations manquantes à la fiche d'enquête, de procéder à l'examen physique des malades, d'effectuer des prélèvements sanguins pour déterminer la créatininémie et la phosphorémie et de procéder enfin à un prélèvement urinaire en vue d'une analyse qualitative par le biais d'une bandelette urinaire (BU). La nouvelle classification de l'histoire naturelle du VHB était utilisée dans ce travail [4]. Pour l'évaluation de la fonction rénale, l'insuffisance rénale (IR) ainsi que la maladie rénale ont été recherchées dans notre étude.

**La définition de l'insuffisance rénale:** était basée sur le calcul par la formule MDRD (Modification of Diet in Renal Disease) du Débit de Filtration Glomérulaire estimé (DFGe) à partir de la créatininémie. Le logiciel MEDICALCUL disponible sur la plateforme Android mobile avait servi à faire ce calcul. La classification de la National Kidney Foundation (NKF) des États-Unis était utilisée dans notre étude [5].

**L'atteinte rénale:** était définie par la présence d'un ou plusieurs des groupes d'anomalies suivants: 1) la positivité au moins à une croix (+) de l'un des paramètres de la BU. Il s'agissait essentiellement de la protéinurie et de la glucosurie;

**L'atteinte glomérulaire définie par la présence simultanée:** a) d'une protéinurie positive: de 1+ à 4+ à la BU; b) et d'un DFGe en dessous de 90 mL/min/1,73m^2^

**L'atteinte tubulaire faisant suspecter un syndrome de Fanconi est évoqué devant:** la présence obligatoire d'une hypophosphorémie associée à au moins 2 autres manifestations de dysfonction tubulaire telles que: a) un DFGe en dessous de 90 mL/min/1,73m^2^ ou une diminution brutale de 25% du DFGe ; b) une protéinurie positive : de 1+ à 4+ ; c) une glucosurie positive: de 1+ à 4+ en absence d'un diabète sucré.

**La valeur normale de la phosphorémie étant comprise entre 25 et 45 mg/L:** l'hypophosphorémie était retenue si celle-ci était inférieure à 25 mg/L ou si on notait une diminution de 5 mg/L par rapport à la phosphorémie au diagnostic ou avant traitement. Les données étaient saisies et enregistrées dans le logiciel Epi-Data version 3.1. L'analyse des données était effectuée dans les logiciels Epi-Data Analysis 2.2.2.182, STATA/SE 13.1 et Open-Epi (Open Source Statistiques Épidémiologiques pour la Santé Publique) 3.01. La comparaison des fréquences était faite à l'aide du test de Chi2 de Pearson ou de Yates selon le cas. La différence était considérée comme statistiquement significative pour un p < 0,05.

## Résultats

Cent cinq (105) patients Ag HBs positif étaient inclus dans notre étude dont 65 sous traitement antiviral contre 40 non traités.

**Caractéristiques de la population d'étude**: sur les 105 patients de notre échantillon d'étude, il y avait une prédominance masculine avec une sex-ratio de 2,88. L'âge moyen était de 42 ± 11 ans avec des extrêmes de 18 et 74 ans. L'antécédent d'hypertension artérielle était présent chez 20% des patients (21/105). La cirrhose était présente au moment du diagnostic chez 19,8% des patients (17/105) et 5,7% (6/105) avaient un diabète. Un patient sur quatre dans notre étude prenait un traitement autre qu'un anti-VHB, soit médical ou traditionnel. Il s'agissait soit d'antihypertenseur chez 60% (15/25) ou des produits de la médecine traditionnelle 36% (9/25). Concernant la classification du VHB, 9,5% (10/105) des patients avaient une infection chronique à VHB contre 5,7% (6/105) d'hépatites chroniques à VHB au diagnostic ou avant l'initiation du traitement. Le reste de la population d'étude n'a pas pu être classé (84,8% ; 89/105) du fait de l'absence de certaines variables (charges virales et aminotransférases) dans les observations médicales. Parmi les 65 patients traités 95,4% (62/65) étaient sous TDF et 4,6% (3/65) sous IFN-PEG. Les patients sous TDF étaient pour la plupart entre 6 et 24 mois de traitement avec un pourcentage cumulatif de 78,5% (n=51).

**Prévalence de l'atteinte rénale chez les PVVHB**: sur les 105 patients de notre population d'étude, 41 patients avaient un DFGe en dessous de 90 mL/min/1,73m^2^ soit une prévalence de l'insuffisance rénale de 39%, IC = [29,7-48,3]. Cette insuffisance rénale était légère (60 ≤ DFGe < 90) chez 34,2% (n=36) et modérée (30 ≤ DFGe < 60) chez 4,8% des patients (n=5). Aucun patient n'avait bénéficié d'un examen qualitatif des urines par BU au diagnostic ou avant traitement. Les résultats présentés dans le Tableau 1 sont ceux de la BU réalisée à l'inclusion dans l'étude. En comparant la phosphorémie et la créatininémie initiale avec ceux à l'inclusion dans l'étude, on note l'apparition d'une hypophosphorémie chez 15,2% (16/105) des patients et une élévation de la créatininémie chez 24 patients (29,1%). L'atteinte tubulaire associant: hypophosphorémie, protéinurie, glucosurie et baisse de DFGe était identifiée chez trois patients 2,9% (3/105). Deux de ces patients présentaient déjà une insuffisance rénale avec un DFGe en dessous de 90 ml/min/1,173m^2^ avant l'inclusion dans l'étude. L'atteinte glomérulaire associant une protéinurie et une diminution du DFGe en dessous de 90 mL/min/1.73m^2^ a été identifiée chez quatre patients 3,8% (4/105). Neuf patients 8,6% (9/105) avaient une protéinurie sans insuffisance rénale. Le type d'atteinte rénale était indéterminé dans le reste des cas 33,3% (35/105). Le Tableau 2 résume l'ensemble des atteintes de la fonction rénale.

**Tableau 1:** bilan rénal des PVVHB à l’inclusion dans l’étude

	Effectif (n)	Fréquence (%)
**Leucocyturie**		
Négatif	101	96,2
1+	4	3,8
**Nitriturie**		
Négatif	102	97,1
1+	2	1,9
2+	1	1,0
**Hématurie**		
Négatif	102	97,1
1+	1	1,0
3+	2	1,9
**Protéinurie**		
Négatif	90	85,7
1+	9	8,6
2+	5	4,8
3+	1	1,0
**Fonction rénale initiale**		
Normale	75	71,4
Insuffisance rénale légère	30	28,6
**Phosphorémie (variation)**		
Normale	81	77,1
Hypophosphorémie	16	15,2
Hyperphosphorémie	8	7,6
**Créatininémie (variation)**		
Normale	81	77,1
Elevée	24	29,1

**Tableau 2:** mécanisme de l’atteinte rénale

	Effectif (n)	Fréquence (%)
**Fonction rénale normale**	64	**-**
Absence d’atteinte rénale	54	84,4
Protéinurie sans I.R	9	14,1
Atteinte tubulaire sans I.R	1	1,5
**Fonction rénale altérée**	41	**-**
Atteinte rénale indéterminée avec I.R	35	85,4
Atteinte tubulaire avec I.R	2	4,9
Atteinte glomérulaire	4	9,7
**Total**	105	**-**

### Facteurs associés à l'atteinte rénale

**Facteurs associés au déclin initial de la fonction rénale chez les PVVHB:** le déclin de la fonction rénale était significativement associé à l'âge supérieur à 50 ans (OR = 0,32 [0,13-0,80], p = 0,0125), l'hypertension artérielle (OR = 0,23 [0,08-0,65], p = 0,0037), à une altération de la fonction rénale initiale (OR = 0,07 [0,02-0,20]; p < 0,0003) et aux co-médications associées (OR = 0,20 [0,07-0,53], p = 0,0007). Le Tableau 3 est un récapitulatif des différents facteurs associés au déclin de la fonction rénale chez les PVVHB.

**Tableau 3:** facteurs associés à l’insuffisance rénale

	Insuffisance rénale		P	OR [IC95%]
	Oui (%)	Non (%)		
**Age (en année)**			**0,0125**	
≤ 50	25 (32,1)	53 (67,9)		0,32 [0,13-0,80]
> 50	16 (59,3)	11 (40,7)		1
**Sexe**			0,2445	
Masculin	33 (42,3)	45 (57,7)		1
Féminin	8 (29,3)	19 (70,4)		0,57 [0,22-1,47]
**HTA**			**0,0037**	
Oui	14 (66,7)	7 (33,3)		1
Non	27 (32,1)	57 (67,9)		0,23 [0,08-0,65]
**Diabète**			**0,4817**	
Oui	1(16,7)	5 (83,3)		1
Non	40 (40,4)	59 (59,9)		3,38 [0,38-30,11]
**Dyslipidémie**			0,2499	
Oui	3 (75,0)	1 (25,0)		1
Non	17 (32,7)	35 (32,7)		0,16 [0,01-1,67]
**I.R avant traitement**			**< 0,0001**	
Oui	24 (80,0)	6 (20,0)		1
Non	17 (22,7)	58 (77,3)		0,07 [0,02-0,20]
**Traitement antiviral**			0,2807	
Oui	28 (43,1)	37 (56,9)		1
Non	13 (32,5)	27 (67,5)		0,63 [0,27-1,45]
**Co-médication associée**			**0,0007**	
Oui	17 (68,0)	8 (32,0)		1
Non	24 (30,0)	56 (70,0)		0,20 [0,07-0,53]

**Facteurs associés à l'évolution de la fonction rénale chez les PVVHB:** en comparant la fonction rénale au diagnostic ou à l'initiation du traitement avec celle à l'inclusion de notre étude, nous avons constaté une dégradation de la fonction rénale chez 21,0% (n = 22) des patients contre une amélioration de la fonction rénale chez 5,7% (n = 6) patients. D'après le Tableau 4, l'âge supérieur à 50 ans (p = 0,002), l'HTA (p = 0,001), une fonction rénale initialement altérée (p = 0,0003) et une co-médication associée (p = 0,0007) influençaient l'évolution de la fonction rénale (défavorablement). Le traitement du VHB ne semblait pas influencer l'évolution de la fonction rénale (p = 0,05) tout comme le diabète, le stade évolutif de la maladie hépatique, la cirrhose, la dyslipidémie et la durée du traitement.

**Tableau 4:** facteurs associés à l’évolution de la fonction rénale chez les PVVHB

	Insuffisance rénale			
	Stabilisation (%)	Dégradation (%)	Amélioration (%)	
**Age (en année)**				**0,002**
≤ 50	64 (82,1)	11 (14,1)	3 (3,8)	
> 50	13 (48,1)	11 (40,7)	3 (11,1)	
**HTA**				**0,001**
Oui	9 (42,9)	10 (47,6)	2 (9,5)	
Non	68 (81,0)	12 (14,3)	4 (4,8)	
**Diabète**				0,776
Oui	5 (83,3)	1 (16,7)	0 (0,0)	
Non	72 (72,7)	21 (21,2)	6 (6,1)	
**Dyslipidémie**				0,256
Oui	2 (50,0)	2 (50,0)	0 (0,0)	
Non	37 (71,2)	9 (17,3)	6 (11,5)	
**Cirrhose**				0,115
Oui	12 (70,6)	2 (11,8)	3 (17,6)	
Non	50 (72,5)	16 (23,2)	3 (4,3)	
**Fonction rénale initiale**				**0,0003**
Normale	58 (77,3)	17 (22,7)	0 (0,0)	
Altérée	19 (63,3)	5 (16,7)	6 (20,0)	
**Traitement**				0,050
Oui	43 (66,2)	16 (24,6)	6 (9,2)	
Non	34 (85,0)	6 (15,0)	0 (0,0)	
**Durée du traitement**				0,741
Moins de 6 mois	7 (87,5)	1 (12,5)	0 (0,0)	
6 à 12 mois	20 (66,7)	8 (26,7)	2 (6,7)	
13 à 24 mois	12 (57,1)	6 (28,6)	3 (14,3)	
Plus de 24 mois	4 (66,7)	1 (16,7)	1 (16,7)	
**Co-médication associée**				**0,0007**
Oui	12 (48,0)	12 (48,0)	1 (4,0)	
Non	65 (81,3)	10 (12,5)	5 (6,2)	

## Discussion

Quarante-et-un (41) patients avaient une insuffisance rénale sur les cent cinq (105) recrutés soit une prévalence de 39%. En absence de données comparatives en population générale au Bénin notre prévalence se rapproche de celle trouvée au Sénégal (pays d'Afrique de l'Ouest comme le Bénin) par Faye *et al*. [6] en 2014 qui était de 37% en zone périurbaine. Dans l'étude HARPE (hepatitis and renal parameters evaluation) [7] évaluant la prévalence des anomalies rénales dans l'hépatite chronique B en France en 2012, les patients avec un DFGe inférieur à 90 mL/min/1.73m2 atteignaient les 55,8%. Cette prévalence élevée de l'insuffisance rénale pourrait s'expliquer non seulement par la taille élevée de l'échantillon utilisé dans cette étude, mais aussi par le fait qu'aucun patient n'y était initié au traitement antiviral. Les résultats auraient été peut-être différents si cette série comportait des patients sous traitement. Shin *et al*. [8] avaient trouvé en 2015 chez les patients de la Samsung Medical Center de Séoul (Corée du sud) une prévalence d'insuffisance rénale largement en dessous de la nôtre : 16,9%. Deux raisons possibles pourraient expliquer cette différence. D'une part les patients de cette étude étaient tous sous analogue nucléot(s)idique, ce qui élimine à priori les cas de néphropathie liée au VHB. D'autre part la méthodologie utilisée dans cette étude définissait l'insuffisance rénale comme une diminution de plus de 25% du DFGe par rapport au DFGe de base. Concernant les facteurs associés au déclin de la fonction rénale, la prise de médicaments autres que les anti-VHB était associée à une atteinte rénale chez les patients de notre étude (p=0,0007). Shin *et al*. [8] avaient noté que la prise de diurétiques était associée à une baisse de la fonction rénale. Certains antihypertenseurs comme les inhibiteurs de l'enzyme de conversion de l'angiotensine (IEC) entraîneraient une insuffisance rénale par diminution de l'irrigation rénale [9].

Le pourcentage d'HTA dans notre étude est similaire à celui de Thu *et al*. [10] qui trouvaient à Bangkok (Thaïlande) en 2015 une HTA chez 23,5% des PVVHB. L'hypertension artérielle, même légère à modérée, est un facteur de risque d'insuffisance rénale chronique, avec une incidence plus importante chez les sujets de race noire [11]. Dans notre série l'HTA était significativement associée au déclin de la fonction rénale (OR=0,23 [0,08-0,65]; p=0,0037). Shin *et al*. [8] avaient fait le même constat (OR=2,03 [1,72-2,39]; p<0,001). Le diabète était trouvé chez 5,7% (6/105) des patients de notre étude, ce qui est similaire au résultat de l'étude HARPE [7]. Cette prévalence du diabète dans notre série est probablement sous-estimée, vu que, nous nous étions contentés de la notion à l'interrogatoire des patients. Rares étaient ceux qui avaient réalisé après leur bilan initial une glycémie à jeun. Le diabète constitue déjà un facteur de risque d'insuffisance rénale chez les sujets non infectés au VHB [12]. Dans notre étude, le diabète n'était pas significativement associé à l'insuffisance rénale (OR=3,38 [0,38-30,11]; p=0,4817). Shin *et al*. [8] avaient démontré le contraire, avec une importante implication du diabète dans le déclin de la fonction rénale chez les PVVHB (OR= 2,80 [2,39-3,27] ; p<0,001). La présence d'une insuffisance rénale au diagnostic ou à l'initiation du traitement antiviral constituait un facteur de risque de déclin de la fonction rénale (p<0,0003). Gara *et al*. [13] ainsi que Shin *et al*. [8] avaient trouvé des résultats similaires. Dans notre population d'étude, 30 patients (28,6%) avaient une insuffisance rénale au diagnostic ou à l'initiation du traitement. Celle-ci avait persisté chez 19 patients (18,1%) et s'était même aggravée chez 5 patients (4,8%) à l'inclusion dans l'étude. Il s'agissait probablement d'une insuffisance rénale chronique qu'un bilan rénal trois mois plus tard ainsi qu'une échographie nous auraient permis de confirmer.

Notons par ailleurs que 6 patients avaient eu leur fonction rénale corrigée à l'introduction du traitement antiviral. Avaient-ils développé une néphropathie liée au VHB qui se serait corrigée après la mise sous TDF ou IFN-PEG ? La réalisation d'une biopsie rénale avec examen anatomopathologique de la pièce de biopsie nous aurait permis de répondre à cette interrogation. Sur les 105 patients de notre étude 65 étaient sous traitement antiviral (61,9%) contre 40 non traités (38,1%). La majorité des patients traités était sous TDF (95,4%). La présence ou non de traitement antiviral n'était pas statistiquement associée à l'insuffisance rénale (OR=0,63 [0,27-1,45], p=0,2887), ni aux différents mécanismes de l'insuffisance rénale (p=0,872) ni à l'évolution de la fonction rénale (p=0,05). L'implication du TDF dans la survenue de l'insuffisance rénale est toujours discutée. Jusqu'en 2010 la sécurité liée à l'usage du TDF était garantie si elle était prescrite en monothérapie chez le patient mono-infecté au VHB. Cependant les récentes observations faites par plusieurs auteurs ont permis d'émettre des réserves quant à l'innocuité de cette molécule. Gara *et al*. [13] trouvaient qu'une dysfonction tubulaire rénale se développait chez 14% des patients suivis sous ADV ou TDF pour une durée de 2 à 9 ans. D'autre auteurs ont calmé ces craintes en concluant que le TDF était sûr pour les PVVHB mais qu'il serait plus qu'important d'instaurer une surveillance de la fonction rénale en présence de patients présentant soit un âge avancé, une HTA, un diabète, une insuffisance rénale sous-jacente, une transplantation hépatique ou rénale et la prise de diurétique ou d'autre médicaments à potentiel néphrotoxique [8, 10, 14]. La majorité des facteurs sus cités ont été identifiés comme associés à l'atteinte rénale dans notre étude. La néphrotoxicité du TDF n'a sans doute pas pu être révélée du fait de la courte exposition des malades de notre étude (2 ans en moyenne). Devant l'implication de l'usage du TDF au long cours dans le déclin de la fonction rénale, une nouvelle molécule le ténofovir alafénamide fumarate (TAF) est proposé [15] et bientôt disponible au Bénin. Les effets secondaires rénaux et osseux du TAF seraient moindres par rapport au TDF. Basit *et al*. aux Etats-Unis d'Amérique en 2017 [15] avaient montré dans une méta-analyse que le TAF était sûr, toléré et aussi efficace que le TDF, mais qu'il faudrait un suivi au long cours pour comparer les éventuels effets secondaires rénaux et osseux à ceux observés avec le TDF. Les dernières recommandations de l'EASL 2017 proposaient déjà le TAF en première intention dans la prise en charge des PVVHB présentant une néphropathie. Les limites de notre étude résident dans: 1) la taille limitée de la population d'étude; 2) l'absence d'évaluation pré-thérapeutique systématique de la protéinurie qui, combinée au DFGe initial, aurait permis de mieux typer l'atteinte rénale avant traitement; 3) l'absence d'évaluation quantitative de la protéinurie (protéinurie des 24 heures ou microalbuminurie). Cela a pu engendrer quelques biais d'information dans notre série.

## Conclusion

La prévalence de l'insuffisance rénale chez les PVVHB était assez élevée (39%). L'âge, l'hypertension artérielle, une insuffisance rénale préexistante et les co-médications étaient identifiés comme des facteurs associés au déclin de la fonction rénale chez les PVVHB.

### Etat des connaissances actuelles sur le sujet

- Le virus de l'hépatite B peut être responsable de manifestations extra-hépatiques rénales, notamment la glomérulonéphrite à VHB et la périartérite noueuse ;
- Le traitement par ténofovir disoproxil fumarate a été associé à de rares cas de dysfonction tubulaire proximale pouvant conduire au syndrome de Fanconi.

### Contribution de notre étude à la connaissance

- La prévalence de l'insuffisance rénale est élevée, de 39% chez les patients suivis pour une hépatite B au CNHU-HKM de Cotonou;
- Les facteurs associés à la survenue d'une insuffisance rénale chez ces patients sont : l'âge supérieur à 50 ans, la présence d'une hypertension artérielle, une fonction rénale initialement altérée, et les co-médications (autres traitements que les anti-VHB);
- Le traitement anti-VHB (TDF ou IFN-PEG) n'est pas associé à la survenue d'une insuffisance rénale.

## Conflits d’intérêts

Les auteurs ne déclarent aucun conflit d’intérêts.
